# Public Health

**Published:** 1884-07

**Authors:** J. McF. Gaston

**Affiliations:** Atlanta, Ga.


THE ATLANTA
MEDICAL AND SURGICAL JOURNAL.

VOL. I. JULY, 1884. No. 5.

### Original Communications.

### PUBLIC HEALTH.

BY J. McF. GASTON, M.D., ATLANTA, GA.

The final cause of the association of men, women and children is undoubtedly the promotion of the good of the whole number forming any society, yet it so turns out that there are incidental features of their close relations within the precinct of a town or city, which bring individual troubles and entail general sufferings that are not encountered by the inhabitants of a sparsely settled locality. The comforts and advantages derived by a community in close proximity and the prompt supply of personal wants by a reciprocity of favors, are counterbalanced to a great extent by the inconveniences growing out of a dense population and the individual neglect of those precautions which should conduce to their mutual benefit and welfare. The philosophy of cities, in which people are brought together for their joint interest, is a matter of moment in a commercial and social aspect, as a part of the great problem of life. But it is from a hygienic standpoint that the population of a city is to be viewed as a whole, and in its relations to the inhabitants of other localities, with reference to the prophylactic measures which are indicated for the promotion of health, as the normal condition of the human race. The aim and end of all hygienic appliances should be the perfection of man's physiological organism, while social, municipal and national health regulations should be directed to the removal of local and general causes of disease.

Among the resolutions adopted April 11th, 1884, by the new State Board of Louisiana, the following are worthy of note:

"*Resolved*, That recognizing the great importance of securing the co-operation of the Board of Health of the States, and of other health associations, wherever situated, and establishing a condition of absolute confidence, it is hereby made the duty of the president and other officers of this board to extend to Boards of Health of other States and other health associations, unrestricted access to the records and health reports of this board, as well as the several quarantine stations, as at the central office of this Board at New Orleans; and it is hereby further made the duty of the president of this Board to make public from day to day, as may be necessary, the condition of the public health; and it is hereby specially required, in the event yellow fever should be introduced into this city or State, to communicate such fact without delay to the exchanges and commercial bodies in New Orleans, and to the Boards of Health of other cities and States.

"*Resolved*, That having thus declared our purposes and the polity of this Board, it is expected that no credence will be given at home or abroad to any reports respecting the state of the public health in this city or State that are not sanctioned or verified by the action of this Board or its duly appointed officers."

But for the saving word "introduced" coupled with another resolution in regard to "the danger that threatens us from the rear" we might rest easy, but there is no provision made for notice of native born yellow fever. As New Orleans is the Mecca of hygienists it must be inferred by the outside world that whatever is, is right. But this indisposition to consider yellow fever as born of the soil and atmosphere of Louisiana is the greatest obstacle to ridding the city of the disease. For one I cannot concur in the dictum that "more liberal views could not have been entertained by any similar body in the country, and a greater proof of sincerity given than that found in the language of these resolutions."

But while the faintest recognition of spontaneous development of yellow fever in New Orleans does not appear in these resolutions of the Board of Health, there is a clear and distinct enunciation of a belief in its being to the manor born in the address of the President at the meeting of the State Board of Health, held April 25th. A correct appreciation of the attitude of other members of this Board will be made when it is understood that "the address was unanimously indorsed by the Board." It would seem that the resolutions adopted April 11th and the address indorsed April 25th had very different objective points, and while other health organizations are to be impressed with the precautions against yellow fever being imported into the city of New Orleans, the State Board of Health is to act upon the conviction "that yellow fever may be called into activity in New Orleans without recent importation or importation at all," and that "this disease appears here under circumstances which baffled every effort of attending physicians and boards of health with their corps of sanitary police to detect the slightest clue to importation."

As this confidential confession of faith in the family circle may serve to give a better insight as to the external and internal policy of the Louisiana State Board of Health, and impress others with the importance of acting upon the assumption of a fact in private, though ignored in public, I would commend the following paragraphs to all whom it may concern, exhorting them at the same time to "trust in providence and keep your powder dry:"

"Yellow fever is due to a specific poison, the existence of which is known only as manifested in man. If intangible, imponderable, unrecognizable to any of the senses, we have no positive knowledge of the essential nature of this poison. Every effort to prevent its appearance and to limit its spread must therefore be purely experimental. We next declare that wherever originated it is communicable, and can be conveyed in the recognized methods along highways of commerce by ships and other carriers of formites. In regard to the transmission of yellow fever it is sometimes impossible to determine the boundary line between contagion, strictly speaking, and infection. There can possibly exist but two sources of its appearance in New Orleans. It must be recently imported or it must develop locally. Being transmissible it can certainly be imported. * * * * * * *

"If yellow fever, to exist here, must be imported or must originate, there are two possible avenues of danger. It matters not what our individual opinions; it is the bounden duty of this board to guard every possible avenue of danger.

"We must prosecute quarantine with a rigor resorting even to non intercourse as though we have no faith in any other measure; while on the other hand we must urge municipal sanitation as though we doubted the absolute efficiency of quarantine.

"It is suggestive that in certain cities formerly devastated by this pestilence, the scourge has ceased coincidently with an improved sanitary system—Boston, New York, Baltimore, Philadelphia, Charleston, S. C. Simple coincidence does not repeat itself five times. Absolute non-intercourse with infected ports can furnish the only positive guarantee against importation. * * * *

"While quarantine is theoretically protection, in practice and tested by the exactions of scientific investigation it is still an experiment, its conclusions not yet absolutely established, we must secure the full benefits offered by its enforcement.

"This matter of responsibility, gentlemen, does not rest solely upon the Board of Health. There are two equally liable: the board on the one hand and the city and State on the other. * * When we undertake to accomplish a $50,000 work with $6,000, and the work of eighty men with ten men, without horse or cart, this is a responsibility which rests upon the city of New Orleans and the State of Louisiana."

Let others look at both sides of this shield of health and profit by the example of New Orleans in the past and in the present neglect of that sage old maxim that "an ounce of prevention is worth a pound of cure." The expenditure of millions in the treatment of yellow fever epidemics, during the last quarter of a century, in the city of New Orleans, has not yet taught her municipal authorities that half the amount spent annually in removing filth from the hidden recesses of the back yards of her inhabitants would secure them against the repetition of these scourges. With a thorough system of drainage and sewerage, accompanied by proper sanitary regulations as to the surroundings of the habitations of the people, there would not be so much necessity for a rigid quarantine around the city of New Orleans or any other seaport town of the United States. The repugnance to the idea of a spontaneous generation of yellow fever in New Orleans has been and continues to be a barrier to expenditures that may not only prevent the local development but secure against the propagation of yellow fever when introduced from other sources.

It is conceded by hygienists generally that there are certain combinations of circumstances requisite for the extension of yellow fever even in those localities most predisposed to it, and the removal of such conditions is the indication in the use of prophylactic measures. All the energies of those in authority and all necessary outlay should be directed towards eliminating this chance of miscarriage, and let it cost what it may, all our cities should be above suspicion in this respect.

None of our Southern towns along highways of commerce can be considered exempt from the inroads of yellow fever during the warmer months of summer, and it behooves all of them to look most sedulously to the complete removal of all offal, garbage or other accumulations that may contaminate the air.

With the experience of past years, no place having even a small population which omits the observance of the strictest sanitary regimen, can presume upon escaping this deadly poison, and even elevations to which in former times it was held that yellow fever never reached have been invaded. The writer has observed cases of yellow fever at an altitude of 2,000 feet above the sea, and more than a hundred miles from the coast line, in Brazil on 23 South latitude; so that, the other conditions favorable to its generation and propagation being present, there is no sort of guarantee afforded by the locality. In view of such well established facts in regard to the enlargement of the domain of yellow fever, every city, town and village in the Southern States should proceed upon the supposition that the population may be exposed to this disease if proper precautions are not taken to secure entirely and completely the cleanliness of every nook and corner of every house and yard, as well as throughout the length and breadth of all the streets. It is a good thing within itself to be rid of filth, and should it be considered a work of supererogation so far as any epidemic is concerned, no family should object to co-operating in a general wholesale cleaning up of premises daily.

In establishing a standard of health for the comparison of different mortuary statistics, the typical population is the family group, and the greater or less number of such groups thrown into close relations in villages, towns and cities, must give the sum total of diseases and deaths for the respective localities. The results of such associations in like places, under like circumstances, furnish a basis for estimating the relative value of hygienic appliances in the different communities, and it is only thus that we can reach a satisfactory conclusion as to the health status of any special population. Evidently there could be no proper comparison between a locality with general conditions of an unfavorable order and one having salutary surroundings by natural position, and hence it is requisite in the estimate of the drawbacks or the advantages of the course of things observed in any given case, to put it into the scale with one similarly situated as to altitude and latitude, so that the accidental concomitants may be duly weighed. In China, Pekin is found to have a death rate of 50 to the thousand inhabitants, owing doubtless to the want of efficient sewerage, lack of water supply, filthiness of the streets and general neglect of sanitary regulations. In Egypt, Cairo has a death rate of 37 per one thousand from the filtering of the sewage into the Nile above the point from which the water is supplied to its large population. The death rate of St. Petersburg is 35 to the thousand; the supply of water from the Neva being contaminated by the sewage which percolates through the soil without sewers. Strange to say, Glasgow and Dublin compare very nearly in their death rate with Bombay, Calcutta and Lisbon, each of these running up to 30 in the thousand of population.

If some of the most populous cities of the world in which drainage and sewerage have been effectually carried out are contrasted with the above named centers of trade in which such hygienic precautions have been disregarded, the advantages of sanitary appliances will be clearly demonstrated in the diminished death rate. After purification by fire and the plague through which London passed many years ago, a most thorough system of drainage was adopted, and, with the gradual experience of passing years, has been so perfected that to-day with nearly four millions of people she presents a death rate of only 20 to the 1,000, having a ratio of births per annum of 34 to the 1,000 of population. Considering the magnitude of the city and poverty of a large proportion of the inhabitants, this result of prophylactic measures on such a stupendous scale is a wonderful triumph of science over difficulties.

In like manner Paris, with over two millions of people, has brought her death rate down to 23 in the 1,000, and Berlin, with over one million of population, and Brussels, which has been styled a miniature Paris, present a death rate of 24 to the 1,000, while Edinburgh only shows 19 to 1,000.

Coming to our enterprising city of New York with over a million inhabitants, it makes an unfavorable impression in the comparison by a death rate of 27 to the 1,000, redeemed by Philadelphia with 19 and Boston with 21 to the 1,000.

It will doubtless strike some with surprise that the best record in vital statistics among the important cities of the world is afforded by St. Louis, with her consolatory small death rate of 11 to the 1,000 and her cheering birth register of 25 annually to every 1,000 of her population, which more than doubles the ratio of deaths.

In the list of gradual increase in deaths to the 1,000 of population, come in order San Francisco 15.8, Chicago 16.5, Cincinnati 17.3, Brooklyn 18.4 and Baltimore 18.5, with a death rate of 21.12 to the 1,000 in Atlanta, upon an average of the past four years.

From the fifth annual report of the Board of Health of the city of Atlanta for the year 1883 we learn that of the total number of deaths there were 443 whites and 644 colored. The annual rate of mortality per thousand among the whites was 14 76; among the colored population 32.20. The number of deaths among persons over five years of age reached 554; white 249, colored 305. The deaths among children under five years of age were 533; white 194, colored 339. Of this number 261 were under one year old; white 102, colored 159. The number of deaths among children under five years of age for the four months May, June, July and August, were 288 against 245 for the other eight months of the year.

"Among the principal causes of death consumption stands charged with 148—white 50, colored 98; pneumonia 75—white 19, colored 54; other acute lung diseases 35—white 19, colored 16; diarrhœal diseases, including diarrhœa, enteritis, dysentery and cholera infantum 235—white 90, colored 145; typhoid fever 79—white 37, colored 42; malarial fever 3—white 1, colored 2; scarlet fever 2—white 1, colored 1; diphtheria 6—white 5, colored 1; rheumatism 1—white. Forty-two deaths were due to accidents and violence—17 white and 25 colored. There were 106 still-born children reported—40 white and 66 colored.

"The greatest mortality among the whites occurred in the month of July, when it reached 55; the lowest in January and December was 22 each. Among the blacks the greatest mortality occurred in the month of July, 89; the lowest in February, 28. The greatest total mortality occurred in the month of July, 144; the lowest in February, 61.

"It will become apparent by an analysis of these statistics that of the total number of deaths 49.03 per cent. occurred among children under five years of age. Consumption caused 13.61 per cent. of all deaths; acute lung diseases, 9.93 per cent.; diarrhœal diseases, 21.61 per cent., and typhoid fever 7.26 per cent.

The same disproportion is shown in the death rate of the two races year after year."

The population of Atlanta being estimated in round numbers at 50,000, of which 30,000 are white and 20,000 colored, it will be noted that with one-third less in population, the blacks have one-third more in the absolute number of deaths, giving the enormous result of more than double the death rate of that in whites, the latter being 14.76, while the former is 32.20 to the 1,000. While the mortality among whites compares favorably with the most salubrious localities, that of the blacks corresponds to the records of the most pestiferous regions of the civilized world.

There is matter here for the serious consideration of the health department and of the municipal authorities of Atlanta, as most assuredly the supposed unfavorable influence of cold upon the negro has not been confirmed in the greater fatality among them in common with the whites during the summer month of July, and in the least number of deaths during February, one of the coldest months. As the greatest difference against the colored ratio exists in the intestinal diseases and pneumonia, it is a fair inference that bad housing and improper alimentation may have had much to do with the result, and although these domestic provisions pertain especially to individuals, yet a thorough system of hygiene cannot ignore the dwellings and food of the people.

In the report it is notable that $611.30 for vaccine virus, and $97.80 for disinfectants and medicine figure in the expenditure, indicating the zeal used in preventive measures, while the sum of $3,057.42 was expended on account of small-pox. The total number of cases was 55, having 17 deaths—11 whites and 6 blacks; this result indicating a greater tolerance of the disease on the part of the latter race, as there were 29 white and 26 colored subjects treated in the hospital and in private quarters.

Though no reference is made to the deaths from small pox in the general report, it is not to be inferred that they are excluded in the death rate per thousand given in the statement of the Board. Small pox not being in any way dependent upon local causes, other than that of contagion, and being temporary in its prevalence, with the effective recourse of vaccination to prevent its propagation, its results do not enter in the regular vital statistics.

It is found that considerable diligence has been used in the removal of offensive matters from the city, and that upon an average for the days of service throughout the year 58 cart loads of garbage and night soil have been taken out daily, while 455 dead animals have been removed from the city.

The inspectors of the two sanitary districts served 2,561 notices upon the inhabitants to abate nuisances of various kinds, showing the want of consideration of individuals for the public welfare. There were 189 cases made in the recorder's court against the violators of the municipal regulations, and fines imposed amounting to $522.00, as an offset to the detriment caused to the public health.

Such a commentary upon personal sanitation should bring home to those in authority the importance of the greatest diligence in hygienic regulations, and it is notorious that what is considered as the business of everybody is nobody's business.

The necessity for a health organization has been recognized by most corporations and is provided for by municipal enactments, yet as a general rule no compensation is set apart for such services, and it is expecting too much of medical men to devise the ways and means for preventing disease and then be called upon to put them into effect at their own cost of time and actual pecuniary outlay. As prophylactic measures are preferable to curative agencies, so those who put into execution a regimen that exempts from disease should be entitled to remuneration rather than those who arrest the progress of a disorder when developed in the body. Pay health officers in preference to physicians, and not limit the annual allowance to a vote of thanks.

The general principle recognized by all is, that impurities of every kind must be combated successfully to secure the normal constitution of the atmosphere; and that it is only requisite to determine the elements that combine to produce such impurities to secure effective measures of sanitation. And it is just here that the great issue exists, between those implicated in the overt act of doing what may directly or remotely prove hurtful to health and those charged with enforcing hygienic regulations. If it were possible to impress the inhabitants of large cities with the danger to themselves personally of a neglect of cleanliness, in all the details of their households, there is no doubt but that each family would cheerfully acquiesce in the inspection of a health officer as often as might be thought necessary to secure them against the inroads of disease. But the fact of the matter is, that people are unwilling to admit in the first place that they are surrounded by filth in their homes, and resent, in the second place, the imputation of having their premises in a condition that requires cleansing. They look upon cleanliness in the light of the person who supposed an individual must be very dirty to require washing every day, and hence contented himself with taking a bath once a month. If a general raking and scraping about their yards is resorted to occasionally, the piles of trash and litter are left in masses where they are supposed to be out of sight, and thus establish foci of corruption, by which the air is charged with noxious emanations and rendered unfit for healthy respiration.

The different elements which enter into putrescence and lead to deleterious transformation of the ambient air need to be ferreted out and put away, or correctives applied which shall serve as disinfectants. It is not that we lack knowledge so much as action is needed in eliminating what is known to be hurtful. Line upon line and precept upon precept have been given by those whose investigations are entitled to the highest consideration, and book hygiene has attained to a high distinction in all the civilized world. But we need men that will take off their coats and roll up their sleeves, with their breeches above their ankles, for wading into the mud and mire of practical scavengering. Talk about aerial poisoning as you may, it comes from the decomposing accumulations about the premises of the rich and the poor who dwell in close proximity, constituting the population of cities. The debris of family supplies, and the deposits of excrementitious matter enter into fermentation and destructive processes of decay, or rot while partially excluded from the free circulation of the atmosphere and the light of the sun, so that gaseous combinations are developed which contaminate the surrounding air and lead to the impairment of the health on the part of the inhabitants. Not that all diseases are to be attributed to local causes of this nature, for there are many zymotic diseases which result from sources of contamination which are totally independent of the baleful consequences of the associations of mankind in cities, yet a vast number of preventable disorders spring directly from sins of omission and commission among the people. In the conglomerated mass of men, women and children entering into the formation of cities, there are conditions independent of the personal regimen that affect the public health, and which belong to the municipal department, to which our consideration should be especially directed in seeking to avert the development of disease amongst the population of any locality.

Knowing full well the difficulties attending all efforts at reform in the established usages of any class of people, it seems more promising to present considerations that may serve to convince all of the evil's by which they are surrounded, than to direct their attention to the measures of relief. The health statistics of different localities vary considerably from circumstances beyond the control of the inhabitants, but the comparative mortality of cities in the same latitude and with similar atmospheric surroundings, depends upon the greater or less attention of the respective populations to sanitary regulations. If a large city is compared with a small town it will be found generally that outside of the prevalence of any epidemic the more populous the place the larger will be the death rate per thousand annually; and if there are marked deviations from this general law controlling the aggregation of human beings, it is most frequently owing to neglect of hygienic precautions; and results from defective houses, impurity of water, faulty drainage and deficient scavengering. In other words, the premature decay of the human race originates for the most part from sources which may be reached and corrected, or from causes that are preventable.

The percentage of deaths to the number of people cannot be taken as a criterion of the healthfulness of a locality in the absolute, as there are many forms of disease which do not terminate fatally. All curable disorders, of whatever nature they may be, swell the sick list of an army, for instance, without adding to the death roll; and the same holds throughout communities that suffer from the lesser ills of life; yet the question recurs as to the extent of impairment to the physical organization and the predisposition to other more serious ailments, from these milder forms of sickness. It is unusual that death accompanies the access of chill and fever, yet the consequences of intermittents are such as to leave the system an easy prey to other forms of disease which end fatally; so that finally the death rate becomes an approximate basis for establishing a standard of health even as to the curable class of diseases. Any section of territory with a considerable population, which presents a constant element of minor diseases must have a higher death rate than a region without such lesser derangements, and the vital forces can only be preserved at their maximum of energy when the organization is exempt from disease in all its shapes and degrees. The end and aim of health regulations should be to remove all the causes of disease so far as may be practicable, and the measures best calculated to prevent the development of the ordinary class of curable disorders, will most generally prove efficient in averting the grosser and more fatal maladies that come in the form of epidemics from time to time.

It is proven by vital statistics that the mortality among large numbers of human beings within certain ages may be calculated with such an approximation to certainty, as to afford a reasonable ground for determining the percentage of deaths in given regions of country, and upon this basis the premiums for life insurance are fixed. A very peculiar phase of this mortuary law is that for periods of five years in a large population, the occurrence of a scourge or pestilence which removes within a brief period a greatly increased population over the general monthly average, there is not found to be a material augmentation of the mean average by the year when the subsequent four years are included in the calculation. An explanation of this fact is afforded by the supposition that many of those already predisposed to disease die in epidemics who would otherwise have succumbed during the subsequent four years, and that hence the whole number of deaths in the course of five-year periods, does not augment from the prevalence of pest or cholera at the beginning of such divisions of time.

The greater or less mortality of different countries or different cities depends for the most part upon factors which are persistent, and tend to maintain the death rate about the same for given spaces of five years in the same locality, yet there have been such revolutions effected by hygienic measures as to reduce the ratio of deaths notably in some of the populous cities of the world within the past quarter of a century, and there is a prospect of a still greater reduction of mortality by the perfection of sanitary appliances under individual co-operation.

It is not so much by words as by actions that the interest of all classes of the community must be manifested, and practice is far better than precept touching prophylactic measures. While grave members of sanitary associations are quoting the sentimental generalizations of Herbert Spencer, that "knowledge, which subserves direct self-preservation by preventing loss of health is of primary importance," and the homily of the Rev. Chas. Kingsley that "the art of keeping one's self alive and well, will in some *more civilized* age and country be considered a necessary element in the school course of every child, just as necessary as reading, writing and arithmetic," it behooves the people to cry aloud against the reality of filth on every hand, and go to work in good earnest as scavengers to rid their homes and yards of contamination.

We do not want lectures on the great benefits of hygienic teaching to the youth of the land, but they must be washed and have clean clothes put on them, the floors of their houses must be scrubbed and their bedding must be renewed from time to time, their food must be sound and nourishing, the air must be fresh and pure which they breathe, the surroundings of their dwellings must be rid of all decaying animal or vegetable accumulations ; in fine, all the causes of physical derangement must be corrected, not by the declarations from high places, but by the practical common-sense procedure of taking the bull by the horns and removing the source of trouble. Away with all fine-spun theories, and give us the plain matter of fact in hygiene, by uprooting all deposits of filth.

